# Increased lymph node yield indicates improved survival in locally advanced rectal cancer treated with neoadjuvant chemoradiotherapy

**DOI:** 10.1002/cam4.2372

**Published:** 2019-06-28

**Authors:** Yaqi Wang, Menglong Zhou, Jianing Yang, Xiaoyang Sun, Wei Zou, Zhiyuan Zhang, Jing Zhang, Lijun Shen, Lifeng Yang, Zhen Zhang

**Affiliations:** ^1^ Department of Radiation Oncology Fudan University Shanghai Cancer Center Shanghai PR China; ^2^ Department of Oncology Shanghai Medical College, Fudan University Shanghai PR China

**Keywords:** locally advanced rectal cancer, lymph node yield, neoadjuvant chemoradiotherapy, prognosis, tumor regression grade

## Abstract

**Purpose:**

It is recommended for colorectal cancer to harvest at least 12 lymph nodes (LNs) during surgery to avoid understaging of the disease. However, it is still controversial whether it is necessary to harvest from locally advanced rectal cancer (LARC) patients who underwent neoadjuvant chemoradiotherapy (neo‐CRT). The impact of lymph node yield (LNY) on prognosis in LARC patients was analyzed.

**Materials/Methods:**

In total, 495 LARC patients who underwent neo‐CRT in 2006‐2015 were analyzed. After examining clinicopathological distribution differences between the LNY subgroups (with the threshold of 12), univariate and multivariate Cox survival analyses were performed. Survival plots were obtained from Kaplan‐Meier analyses. Similar subgroup analyses were performed according to the tumor regression grade (TRG) and metastatic status of post‐operational LNs.

**Results:**

Of the 495 patients, 287 (57.98%) had an LNY of less than 12. Nearly no significant clinicopathological difference was found between the LNY subgroups, including the TRG scores. Multivariate survival analysis demonstrated that at least 12 LNs examined was an independent prognostic feature of good overall survival (OS), disease‐free survival (DFS), and distant metastasis free survival (DMFS), but not local recurrence free survival (LRFS). However, in the subgroup analyses, no association was found between LNY and prognosis in patients with good TRG scores (0‐1) or negative LNs.

**Conclusions:**

For LARC patients treated with neo‐CRT, an LNY of at least 12 indicated an improved survival. Decreased LNY was not related to better tumor regression. It suggests that a sufficiently high LNY is still required, especially in those with a potentially poor tumor response.

## 1 INTRODUCTION

Colorectal cancer has the third highest incidence and second highest mortality in the world.^1^ It is recommended for colorectal cancer to harvest at least 12 lymph nodes (LNs) during surgery to avoid understaging of the disease. For locally advanced rectal cancer (LARC), the standard treatment nowadays is neoadjuvant chemoradiotherapy (neo‐CRT) followed by total mesorectal excision (TME).^2^, ^3^, ^4^ However, patients who received neo‐CRT were shown to have a lower number of lymph node yield (LNY) compared with patients treated without neo‐CRT.^5^, ^6^ Some studies have shown that an LNY of less than 12 indicated better tumor regression and better survival.^7^, ^8^, ^9^, ^10^ However, other studies have refuted this conclusion.^11^, ^12^, ^13^ Thus, it is controversial whether the threshold of 12 is applicable to LARC patients after neo‐CRT. In this study, we investigated characteristics associated with LNY, and examined associations among LNY, tumor regression grade, and survival in LARC patients.

## 2 PATIENTS AND METHODS

### 2.1 Study population

Study population between January 2006 and March 2015, we identified 550 consecutive LARC (cT3‐4/N+) patients who underwent neo‐CRT followed by TME surgery at Fudan University Shanghai Cancer Center. All LARC patients received intensity modulated radiation therapy (IMRT, 45‐55Gy, 25 Fractions, 1.8‐2.0Gy per fraction) with concurrent capecitabine‐based chemotherapy. After neo‐CRT, they received 0‐3 cycles of interval chemotherapy (Xeloda, oral capecitabine 1000 mg/m^2^ twice daily on days 1‐14 every 3 weeks, or XELOX, intravenous oxaliplatin 130 mg/m^2^ on day 1 plus oral capecitabine 1000 mg/m^2^ twice daily on days 1‐14 every 3weeks). Finally, surgeries were performed, including anterior resection (AR), abdominal‐perineal resection (APR), and Hartmann surgery, followed by about 4‐8 cycles of adjuvant chemotherapy (XELOX, intravenous oxaliplatin 130 mg/m^2^ on day 1 plus oral capecitabine 1000 mg/m^2^ twice daily on days 1‐14 every 3 weeks). All patients were followed up every 3 months in the first 2 years. The interval was every 6 months in the next 3 years and once every year after the fifth year. The content of follow‐up contained physical examination (especially the digital rectal examination), colonoscopy, laboratory, and imaging examinations (pelvic MRI, and chest and abdomen CT). The success rate of follow‐up was 92.0%.

Only patients who were pathologically proved as rectal adenocarcinoma, had complete pathological files, an adequate follow‐up were included. The patients with other primary malignancies or distant metastases at diagnosis were excluded from the cohort. Patients who had an interval from the completion of radiation to surgery greater than 16 weeks were also excluded. Finally, there were 495 patients met the criteria.

### 2.2 Data collection

Baseline characteristics, therapeutic process, and pathologic files of all patients were reviewed seriously. Gender, age at diagnosis, and distance from the anus were recorded. Clinical stages (cT and cN) were evaluated by MRI specific for rectum. Details of therapeutic process including neo‐CRT, interval chemotherapy, surgery procedure, adjuvant chemotherapy were obtained from the medical histories. The number of LNY was obtained from the patients’ pathological reports. Other pathological features were also collected accurately, including tumor regression grade (TRG), differentiation grade, post‐operational invasion depth (ypT) and positive LNs (ypN), tumor deposits (TDs), neural invasion, vascular invasion, and circumferential resection margins (CRM) invasion, etc. TRG score was evaluated according to the eighth edition of AJCC (American Joint Committee on Cancer) Cancer Staging Manual (0, complete regression with no residual cancer cell; 1, almost complete regression with only one single residual cancer cell or a cluster of cancer cells; 2, moderate regression with many residual cancer cells; 3, minimal regression with nearly no cancer cells killed). The dates of local recurrence, distant metastasis, and death were recorded to calculate the survival times.

### 2.3 Statistical Methods

Continuous data were presented as medians and ranges, and categorical data were presented as frequencies. Clinicopathological distribution differences were compared between the LNY subgroups (with the threshold of 12) using chi‐square test. Associations between the LNY subgroups and survival times (overall survival [OS]; disease‐free survival [DFS]; local recurrence free survival [LRFS]; distant metastasis free survival [DMFS]) were analyzed through univariate and multivariate Cox proportional hazards regression analyses. Survival plots were obtained from Kaplan‐Meier analyses, and two curves was compared using log‐rank test. Then, similar survival analyses were done according to the grade of tumor response (TRG score 0‐1 subgroup and 2‐3 subgroup) and the metastatic status of LNs (LN‐negative subgroup and LN‐positive subgroup). The multivariate Cox analyses included variables which tended to be significant (*P* < 0.1) in univariate analyses or with important clinical significance. Value of *P* < 0.05 was considered as the threshold of significance. SPSS software (SPSS 22.0, Inc, Chicago, IL) was used to conduct the above analyses. Also, forest plots were made using Microsoft Office Excel 2016 (Microsoft Company, Redmond, WA).

## 3 RESULTS

### 3.1 Patient characteristics

In this study, 495 LARC patients were retrospectively analyzed. Multiple characteristics are presented in Table 1. Of the 495 patients, 335 (67.7%) were 50 years and older. A total of 71.1% (352 of 495) of the patients were men and 28.9% (143 of 495) were women. Most of the patients (78.2%, 387 of 495) were classified as cT3. In addition, 44.6% (221 of 495) and 43.0% (213 of 495) of the patients were classified as cN1 and cN2, respectively. Of 495 the patients, 354 (71.5%) received operations within 60 days after the completion of neo‐CRT. The proportions of the three operation procedures were 39.2% (194 of 495) for AR, 53.9% (267 of 495) for APR, and 6.9% (34 of 495) for Hartmann surgery. In addition, 64.6% (320 of 495) of the patients received interval chemotherapy and 92.9% (460 of 495) of the patients received adjuvant chemotherapy. Pathological files revealed that 21.2% (105 of 495) of the patients achieved pathological complete response (pCR, TRG = 0, and ypT0N0TD0), and most of the patients (70.1%, 347 of 495) had negative LNs after operation. In addition, TDs were found in 17.8% (88 of 495) of the patients. Of the 495 patients, 53 (10.7%), 32 (6.5%), and 3 (0.6%) exhibited positive invasion of nerves, vessels, and CRM, respectively.

**Table 1:** Patients’ characteristics stratified by the LNY subgroups

Characteristics, No. (%)	All (N = 495)	LNY < 12 (N = 287)	LNY ≥ 12 (N = 208)	*P*‐value
Age (y)				0.130
< 50	160 (32.3)	85 (29.6)	75 (36.1)	
≥50	335 (67.7)	202 (70.4)	133 (63.9)	
Gender				0.855
Male	352 (71.1)	205 (71.4)	147 (70.7)	
Female	143 (28.9)	82 (28.6)	61 (29.3)	
cT				0.825
T2	11 (2.2)	7 (2.4)	4 (1.9)	
T3	387 (78.2)	226 (78.7)	161 (77.4)	
T4	97 (19.6)	54 (18.8)	43 (20.7)	
cN				0.855
N0	61 (12.3)	37 (12.9)	24 (11.5)	
N1	221 (44.6)	129 (44.9)	92 (44.2)	
N2	213 (43.0)	121 (42.2)	92 (44.2)	
Distance from anus (cm)				0.874
≤5	290 (58.6)	169 (58.9)	121 (58.2)	
>5	205 (41.4)	118 (41.1)	87 (41.8)	
Radiation dose (Gy)				0.219
≤50	396 (80.0)	235 (81.9)	161 (77.4)	
>50	99 (20.0)	52 (18.1)	47 (22.6)	
Interval chemotherapy				0.104
No	175 (35.4)	110 (38.3)	65 (31.3)	
Yes	320 (64.6)	177 (61.7)	143 (68.8)	
Interval time				0.008
<60	354 (71.5)	192 (66.9)	162 (77.9)	
≥60	141 (28.5)	95 (33.1)	46 (22.1)	
Surgical procedure				0.717
APR	267 (53.9)	153 (53.3)	114 (54.8)	
AR	194 (39.2)	116 (40.4)	78 (37.5)	
Hartmann	34 (6.9)	18 (6.3)	16 (7.7)	
Adjuvant chemotherapy				0.544
No	35 (7.1)	22 (7.7)	13 (6.3)	
Yes	460 (92.9)	265 (92.3)	195 (93.8)	
Differentiation grade				0.721
Low	65 (13.1)	35 (12.2)	30 (14.5)	
Middle	203 (41.0)	120 (41.8)	83 (40.1)	
High	16 (3.2)	11 (3.8)	5 (2.4)	
Unknown	210 (42.5)	121 (42.2)	89 (43.0)	
TRG				0.446
0	105 (21.2)	59 (20.6)	46 (22.1)	
1	133 (26.9)	85 (29.6)	48 (23.1)	
2	222 (44.8)	124 (43.2)	98 (47.1)	
3	35 (7.1)	19 (6.6)	16 (7.7)	
ypT				0.174
T0	105 (21.2)	60 (20.9)	45 (21.6)	
T1	20 (4.0)	12 (4.2)	9 (4.3)	
T2	138 (27.9)	91 (31.7)	46 (22.1)	
T3	203 (41.0)	110 (38.3)	93 (44.7)	
T4	29 (5.9)	14 (4.9)	15 (7.2)	
ypN				0.264
N0	347 (70.1)	202 (70.4)	145 (69.7)	
N1	100 (20.2)	62 (21.6)	38 (18.3)	
N2	48 (9.7)	23 (8.0)	25 (12.0)	
TD				0.155
Negative	407 (82.2)	230 (80.1)	177 (85.1)	
Positive	88 (17.8)	57 (19.9)	31 (14.9)	
Vascular invasion				0.869
Negative	463 (93.5)	268 (93.4)	195 (93.8)	
Positive	32 (6.5)	19 (6.6)	13 (6.3)	
Neural invasion				0.504
Negative	442 (89.3)	254 (88.5)	188 (90.4)	
Positive	53 (10.7)	33 (11.5)	20 (9.6)	
CRM invasion				0.386
Negative	492 (99.4)	286 (99.7)	206 (99.0)	
Positive	3 (0.6)	1 (0.3)	2 (1.0)	

Abbreviations: CRM, circumferential resection margin; LNY, lymph node yield; TD, tumor deposit; TRG, tumor regression grade

In terms of the count of LNY, greater than 11 LNs were examined in 42.0% of patients (208 of 495). We separated the patents into an LNY < 12 subgroup and an LNY ≥ 12 subgroup. Distribution of clinicopathological features based on the LNY subgroups is also presented in Table 1. No clinicopathological difference between the two LNY subgroups was found in sex, age, cT, ypT, cN, ypN, distance from the anus, treatment procedures, differentiation grade, TD, neural invasion, vascular invasion, and CRM invasion (all P＞0.05). Also, the LNY subgroups had no association with TRG scores (*P* = 0.446), which indicated that less than 12 of LNs harvested was not a predictor of better response. However, the interval time subgroups divided by 60 days was negatively related to the LNY subgroups (*P* = 0.008). The underlying mechanism was probably that the longer interval time brought more tissue regression and fibrosis, finally led to lower number of LNY.

### 3.2 Prognostic significance of LNY in all LARC patients

**Figure 1:**
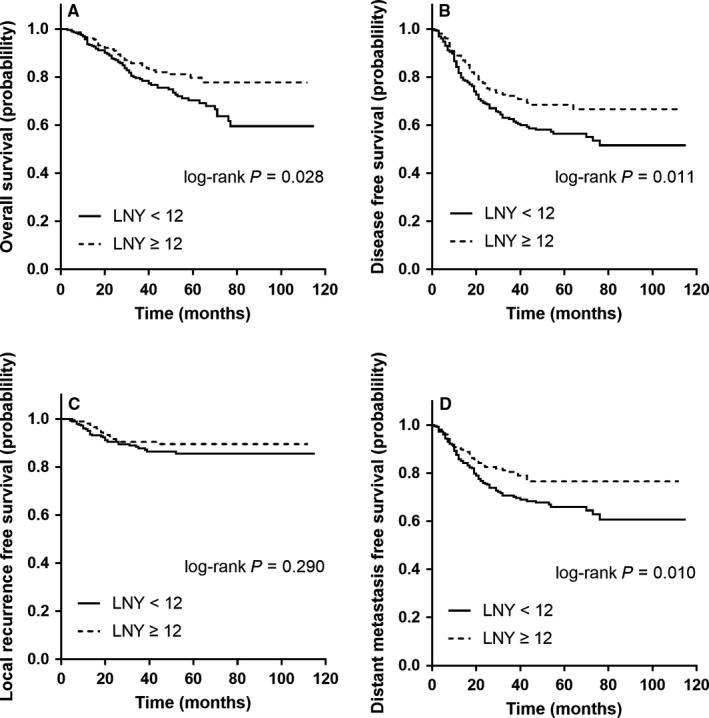
Kaplan‐Meier curves of survival times stratified by the lower and higher LNY subgroups

Compared with the LNY < 12 subgroup, univariate Cox survival analyses found that the LNY ≥ 12 subgroup had better OS, DFS and DMFS, not LRFS. Survival curves were obtained from Kaplan‐Meier analysis (Figure 1A‐D; log‐rank test P values: OS 0.028, DFS 0.011, LRFS 0.290, DMFS 0.010). Associations between multiple clinicopathological variables and survival times examined through univariate analyses were also shown in Table .

**Figure 2:**
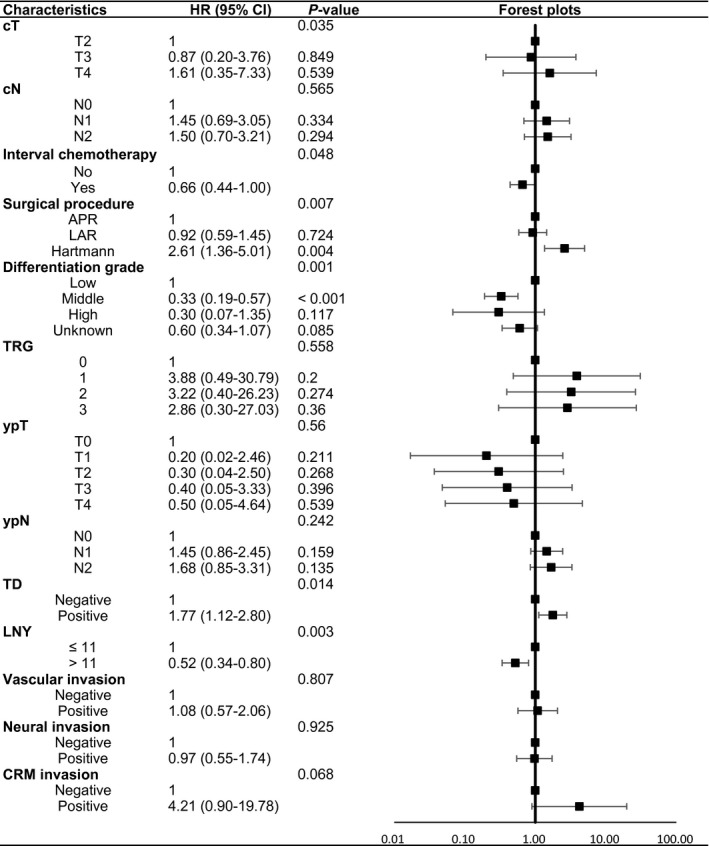
Forest plots of adjusted HRs and 95% CIs in relation to OS

**Figure 3:**
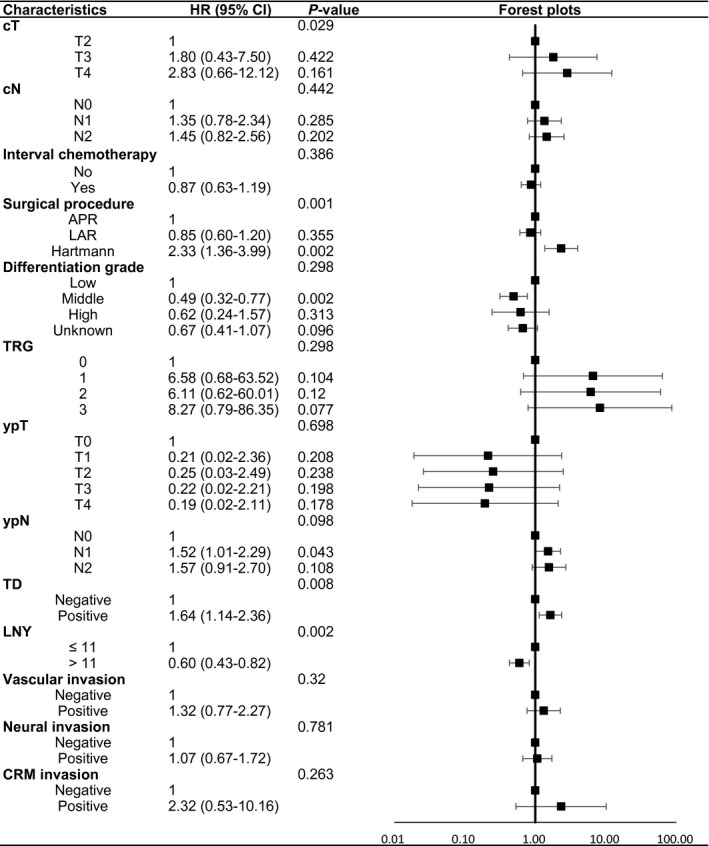
Forest plots of adjusted HRs and 95% CIs in relation to DFS

Next, variables which tended to be significant (*P* < 0.1) in univariate analyses or with important clinical significance were included into multivariate Cox proportional hazard regression models. After the adjustment for multiple clinicopathological factors, patients with an LNY of at least 12 were still associated with decreased relative risk of distant metastasis with an adjusted hazard ratio (HR) of 0.58 (95% confidence interval (95% CI) 0.40‐0.85, *P* = 0.005), but had no association with local recurrence rate (HR 0.60, 95% CI 0.33‐1.09, *P* = 0.093). Finally, at least 12 LNs examined was proved to be an independent predictor of good DFS (HR 0.60, 95% CI 0.43‐0.82, *P* = 0.002) and OS (HR 0.52, 95% CI 0.34‐0.80, *P* = 0.003). The adjusted HRs and 95% CIs of OS and DFS were displayed in Figures 2 and 3 using forest plots. In addition, cT, operation procedures, differentiation grade, and tumor deposit were independently related to OS. For DFS, cT, surgical procedures, and tumor deposit were independent prognostic factors. For LRFS, factors including cT, TRG score, tumor deposit, and CRM remained significant. Also, for DMFS, only TRG scores had independent prognostic significance. All the results of multivariate Cox survival analyses could be looked up in Table .

### 3.3 Prognostic significance of LNY in postoperative LN‐negative or LN‐positive patients

**Figure 4:**
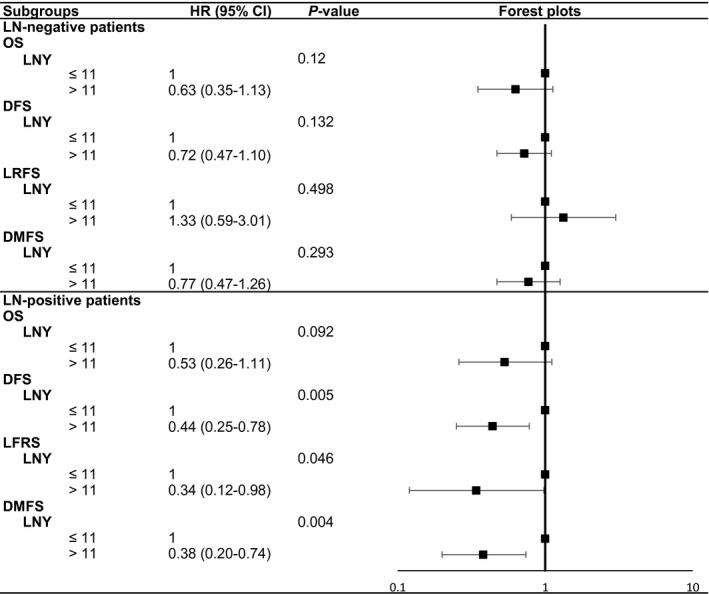
Forest plots of adjusted HRs and 95% CIs in relation to survival times in patients with different metastatic status of LNs

Patients with positive and negative postoperative LNs had an average of 10.73 ± 5.17 and 10.19 ± 5.23 LNs examined (*P* = 0.293). For postoperative LN‐negative patients, no association was found between the LNY subgroups and four survival times in multivariate Cox survival analyses, showing nonsignificant HRs and 95% CIs (all *P* > 0.05, Figure 4).

For the postoperative LN‐positive subgroup, Kaplan‐Meier plots showed better LRFS (*P* = 0.041), DMFS (*P* = 0.007), and DFS (*P* = 0.015) in LNY ≥ 12 patients compared with LNY < 12 patients, not including OS (*P* = 0.162). Furthermore, the results of multivariate Cox survival analyses also confirmed this conclusion. The LNY ≥ 12 subgroup remained the benefit for LRFS with the adjusted HR of 0.34 (95% CI 0.12‐0.98, *P* = 0.046) and DMFS with the adjusted HR of 0.38 (95% CI 0.20‐0.74, *P* = 0.004), as well as the DFS (HR 0.44, 95% CI 0.25‐0.78, *P* = 0.005). Unfortunately, these benefits did not translate into better OS (HR 0.53, 95% CI 0.26‐1.11, *P* = 0.092). The adjusted HRs and 95% CIs of the four survival times were displayed using forest plots (Figure 4).

### 3.4 Prognostic significance of LNY in the good or poor tumor response subgroups

TRG score is widely used for evaluating the grade of tumor response to neo‐CRT and is an important predictor of following prognosis. Patients with TRG score 0‐1 represents good tumor response and patients with TRG score 2‐3 represents poor tumor response. There were 10.21 ± 5.08 and 10.49 ± 5.35 LNs harvested in patients with good and poor tumor regression (*P* = 0.554).

**Figure 5:**
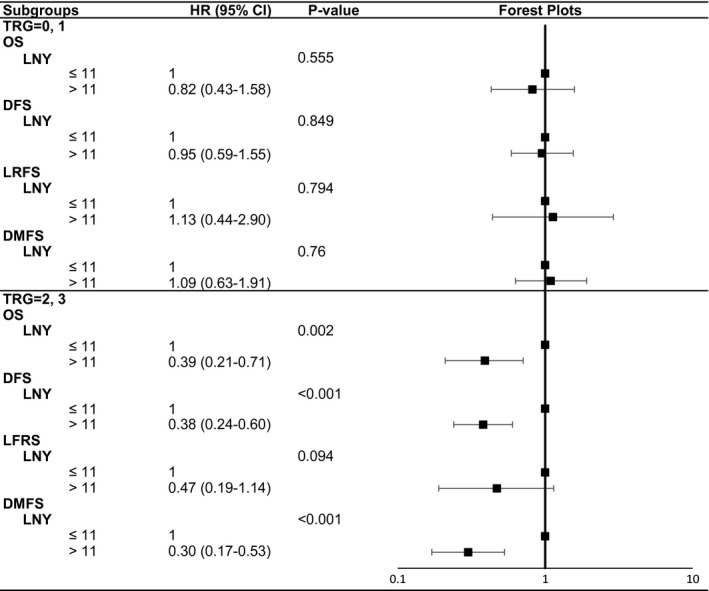
Forest plots of adjusted HRs and 95% CIs in relation to survival times in patients with good or poor tumor response

For the subgroup with lower TRG scores (0‐1), multivariate Cox survival analyses revealed that patients in the two LNY subgroups had similar OS, DFS, LRFS, and DMFS with non‐significant 95% CIs of HRs (all *P* > 0.05, Figure 5).

For the subgroup with higher TRG scores (2‐3), survival analyses with the Kaplan‐Meier method demonstrated that patients with at least 12 LNs harvested showed longer OS (*P* = 0.039), DMFS (*P* < 0.001), and DFS (*P* = 0.002) than patients with less than 12 LNs, but did not have significantly different LRFS (*P* = 0.302). Furthermore, multivariate Cox survival analyses verified the same findings. After the adjustment for other variables, the LNY ≥ 12 subgroup still had improved DMFS and DFS than the LNY < 12 subgroup, with the adjusted HR of 0.30 (95% CI 0.17‐0.53, *P* < 0.001) and 0.38 (95% CI 0.24‐0.60, *P* < 0.001). Although the LNY ≥ 12 subgroup did not show increased LRFS (HR 0.47, 95% CI 0.19‐1.14, *P* = 0.094), they could still achieve an improved OS (HR 0.39, 95% CI 0.21‐0.71, *P* = 0.002). Forest plots of Figure 5 also showed the adjusted HRs and 95% CIs of the above four survival times.

## 4 DISCUSSION

According to present clinical guidelines, it is necessary to harvest at least 12 LNs in the operation of colorectal cancer. Inadequate LNY may result in tumor understaging, increasing the risk of tumor progression, and leading to poorer survival times.^5^, ^14^

The LNY may be influenced by many factors, such as patients’ anatomical and clinicopathological factors, the surgical operation process, and the pathological techniques of lymph node harvest.^15^, ^16^, ^17^, ^18^ It was reported that patients with higher body mass index (BMI), older age, lower comorbidity scores, and more distally located cancers were more likely to have an decreased LNY.^6^, ^13^ It is worth noting that the current guidelines for LNY are more suitable for colon cancers than rectal cancers. The unique anatomical configuration of rectal cancer and the small size of LNs in the mesorectum make it more difficult to retrieve LNs than colon cancer.

Furthermore, neoadjuvant therapy is another factor that could affect the number of LNY. For LARC patients, it is recommended to receive neo‐CRT followed by TME surgery,^2^, ^3^, ^4^ which could significantly decrease the rate of local recurrence.^19^ Several studies demonstrated a decrease in LNY in patients treated with neo‐CRT.^5^, ^6^, ^16^, ^20^, ^21^, ^22^ In 2017, Mechera et al^23^ retrospectively analyzed 34 articles (including 37 datasets) and found that patients with neo‐CRT had a mean reduction of 3.9 LNs and an average reduction of 0.7 in harvested positive LNs compared with patients without neo‐CRT. The mechanism behind this phenomenon may be radiation‐induced fibrosis, tissue shrinkage, lymphocyte depletion, stroma atrophy, and adipocyte replacement,^24^, ^25^ which make it difficult to detect LNs during pathological examinations. Moreover, a longer time interval between neo‐CRT and TME surgery may lead to a reduced LNY because of the development of more stromal fibrosis.

Thus, it is debated whether it is still necessary to harvest at least 12 LNs for LARC patients who received neo‐CRT. Also, the associations among LNY, tumor response, and survival in those patients are uncertain. Some studies have shown that less than 12 LNs harvested indicated good tumor response.^7^, ^8^, ^9^, ^10^ Gurawalia et al^26^ identified 364 rectal cancer patients between 2010 and 2014, of whom 91 were treated with neoadjuvant treatment. Patients with less than 12 LNs harvested were more likely to achieve pCR (40% vs. 26%, *P* < 0.05) and lower TRG scores (*P* < 0.05). Recently, a study by Bustamante‐Lopez et al^27^ found that within multiple clinicopathological features, only pCR remained a significant association with less than 12 LNs in multivariate analyses (*P* = 0.002).

In addition to tumor regression grade, many studies have examined whether this good tumor response could translate into a better prognosis. Damin et al^9^ and Persiani R et al^10^ both verified that after neo‐CRT, the count of LNY was inversely correlated with tumor response. However, lower LNY did not indicate better OS or DFS. De Campos‐Lobato et al^7^ included 237 LARC patients after neo‐CRT and found that the LNY < 12 group had higher rate of pCR (36% vs. 19%, *P* = 0.01) and lower rate of local recurrence, but did not affect distant metastasis. Kim et al^28^ analyzed 1,332 patients, of whom 433 (32.8%) patients received neo‐CRT. Good tumor regression was not only related to lower number of total LNY, but also positive to LNs. Of all the patients, however, the LNY < 12 group had a significantly better 3‐year DFS than the LNY ≥ 12 group only in those with good tumor response (*P* = 0.030).

Thus, the abovementioned studies suggested that for LARC patients treated with neo‐CRT, LNY with a threshold of 12 was not appropriate as a qualification for adequacy of LNY. A series of studies have attempted to find the appropriate threshold of LNY. Hall et al^29^ and Han et al^30^ proved that an at least eight LNs should be harvested to achieve accurate staging. The LNY thresholds of seven and nine were reported in studies by Tsai et al^21^ and Raoof et al^31^.

In contrast, there were many large sample studies in favor of at least 12 LNs harvested. Lykke et al^11^ retrospectively analyzed 6,793 Danish rectal cancer patients treated with or without neo‐CRT between 2003 and 2011 and found that an LNY ≥ 12 was more likely to have better OS, irrespective of neoadjuvant treatment. In 2016, a study by Xu et al^12^ retrospectively analyzed 25,447 rectal cancer patients from the 2006‐2011 National Cancer Database. 62% of them underwent neo‐CRT, and 32% finally obtained decreased LNY. For patients without neo‐CRT, decreased LNY could increase the mortality HR by 18%, when controlled for other clinicopathological factors. Also, this increased mortality HR will be 20% for those with neo‐CRT. Another study with a larger sample size was performed by Cox et al.^13^ Of 38,363 patients, 76% received neo‐CRT. They also demonstrated that increased LNY was related to improved survival for up to 12 LNs harvested, regardless of treatment with (HR 0.79, *P* < 0.0001) or without (HR 0.88, *P* = 0.04) neo‐CRT. These studies showed that at least 12 LNs harvested indicated a good prognosis for rectal cancers regardless of neo‐CRT treatment status. Evaluating fewer than 12 LNs in those after neo‐CRT may lead to inferior survival and understating the disease.

Given the decreased LNY after neo‐CRT, Degiuli et al^32^ focused on clinical significance of the complete absence of LNs harvested (ypNnull). Interestingly, ypNnull patients exhibited the worst prognosis comparing to both ypN0 and ypN + patients, which was contrary to several other articles.^33^, ^34^

The findings of our study were consistent with the latter articles. We found that there were no associations among LNY and multiple clinicopathological variables. In particular, decreased LNY was not related to better TRG (*P* = 0.446). Furthermore, multivariate survival analyses showed that at least 12 LNs harvested indicated better prognosis in terms of OS, DFS, and DMFS independently of other patients’ characteristics. Next, we did further analyses in the postoperative LN and TRG subgroups. Survival benefit of the LNY ≥ 12 group was only demonstrated in patients with positive LNs or poor tumor response (TRG = 2‐3). Thus, we recommend retrieving at least 12 LNs to avoid understaging the disease in LARC patients who underwent neo‐CRT, particularly in those with a higher possibility of positive LNs and poor tumor response.

There are some limitations to our study. First, we only focused on the count of LNY and did not explore its pathological features. Even the negative LNs may change substantially after neo‐CRT, with uncertain clinical significance. Second, we did not attempt to investigate new parameters other than pN status to provide a more accurate prediction of prognosis, such as lymph node ratio (LNR), which was studied in many studies.^35^, ^36^, ^37^, ^38^, ^39^ In addition, it should be taken into consideration that the count of LNs harvested varied according to different pathological materials and techniques. Most of the previous studies (including ours) only analyzed the number or status of LNs and did not describe how these LNs were retrieved and recognized because most researchers are clinicians, not pathologists. Dias et al^40^ provided new perspectives about the underlying problems of decreased LNY in patients treated with neo‐CRT. They proved that Carnoy's solution increased the number of LNs harvested compared with formalin and reduced the rate of inadequate LNY. Some articles have even reported that thorough specimen analysis could obtain higher LNY compared with routine analysis, which indicated that neo‐CRT might not reduce LN count.^41^, ^42^, ^43^, ^44^, ^45^ This finding suggested that for patients undergoing neoadjuvant therapy, the materials and methods for harvesting LNs should be improved to avoid inadequate LNYs.

In conclusion, for LARC patients who underwent neo‐CRT, at least 12 LNs harvested was an independent good prognostic factor and no more than 12 LNs harvested did not indicate good tumor response. We suggested that it is still necessary to obtain a sufficiently high LNY (at least 12) for LARC patients who underwent neoadjuvant chemoradiotherapy, especially for those with a potentially poor tumor response. In addition, improved materials and methods for harvesting LNs are needed.

## CONFLICT OF INTEREST

The authors have no conflicts of interest.

## AUTHOR CONTRIBUTIONS

Conceptualization: Yaqi Wang, Zhen Zhang. Data curation: Yaqi Wang, Menglong Zhou. Formal analysis: Lifeng Yang. Funding acquisition: Zhen Zhang. Investigation: Jianing Yang, Wei Zou. Methodology: Menglong Zhou, Xiaoyang Sun. Project administration: Lijun Shen. Resources: Jianing Yang, Wei Zou. Software: Zhiyuan Zhang. Supervision: Lifeng Yang. Validation: Lijun Shen, Lifeng Yang. Visualization: Jing Zhang, Zhiyuan Zhang. Writing—original draft: Yaqi Wang, Menglong Zhou. Writing ‐ review and editing: Yaqi Wang, Zhen Zhang.
