# Supplementary material for: Increased lymph node yield indicates improved survival in locally advanced rectal cancer treated with neoadjuvant chemoradiotherapy
Source: Cancer Med. 2019 Jun 28;8(10):4615–25. doi: 10.1002/cam4.2372 (PMC6712464; doi:10.1002/cam4.2372)
Supplement: Supplementary file 1 [file CAM4-8-4615-s001.docx]

**Supplemental Materials**

**Supplemental Table 1. Univariate Cox analyses in relation to survival times**

| Characteristics | OS | | DFS | | LRFS | | DMFS | |
| --- | --- | --- | --- | --- | --- | --- | --- | --- |
|  | HR^#^ 95% CI | P | HR^#^ 95% CI | P | HR^#^ 95% CI | P | HR^#^ 95% CI | P |
| Age (y) |  | 0.671 |  | 0.503 |  | 0.340 |  | 0.441 |
| < 50 | 1.00 |  | 1.00 |  | 1.00 |  | 1.00 |  |
| ≥ 50 | 0.92 (0.62-1.36) |  | 1.11 (0.81-1.53) |  | 0.77 (0.44-1.33) |  | 1.16 (0.80-1.67) |  |
| Gender |  | 0.244 |  | 0.623 |  | 0.750 |  | 0.464 |
| Male | 1.00 |  | 1.00 |  | 1.00 |  | 1.00 |  |
| Female | 0.77 (0.49-1.20) |  | 1.09 (0.79-1.50) |  | 0.91 (0.49-1.67) |  | 1.15 (0.79-1.66) |  |
| cT |  | 0.041 |  | 0.050 |  | 0.028 |  | 0.343 |
| T2 | 1.00 |  | 1.00 |  | 1.00 |  | 1.00 |  |
| T3 | 0.97 (0.24-3.94) | 0.963 | 1.88 (0.47-7.61) | 0.374 | 7596  (< 0.001, > 200) | 0.920 | 2.87 (0.40-20.60) | 0.294 |
| T4 | 1.69 (0.40-7.09) | 0.475 | 2.78 (0.67-11.50) | 0.157 | 16708  (< 0.001, > 200) | 0.914 | 3.53 (0.48-25.98) | 0.215 |
| cN |  | 0.009 |  | 0.009 |  | 0.020 |  | 0.014 |
| N0 | 1.00 |  | 1.00 |  | 1.00 |  | 1.00 |  |
| N1 | 1.36 (0.68-2.73) | 0.384 | 1.30 (0.77-2.22) | 0.327 | 1.29 (0.43-3.83) | 0.647 | 1.07 (0.59-1.94) | 0.830 |
| N2 | 2.26 (1.15-4.43) | 0.018 | 1.92 (1.14-3.22) | 0.014 | 2.67 (0.94-7.55) | 0.064 | 1.75 (0.98-3.11) | 0.057 |
| Distance from anus (cm) |  | 0.346 |  | 0.829 |  | 0.457 |  | 0.834 |
| ≤ 5 | 1.00 |  | 1.00 |  | 1.00 |  | 1.00 |  |
| > 5 | 1.20 (0.82-1.75) |  | 1.03 (0.77-1.39) |  | 0.81 (0.46-1.42) |  | 1.04 (0.74-1.47) |  |
| Radiation dose (Gy) |  | 0.842 |  | 0.614 |  | 0.943 |  | 0.382 |
| ≤ 50 | 1.00 |  | 1.00 |  | 1.00 |  | 1.00 |  |
| > 50 | 0.96 (0.61-1.49) |  | 0.91 (0.63-1.31) |  | 0.98 (0.50-1.90) |  | 0.82 (0.53-1.27) |  |
| Interval chemotherapy |  | 0.002 |  | 0.067 |  | 0.671 |  | 0.175 |
| No | 1.00 |  | 1.00 |  | 1.00 |  | 1.00 |  |
| Yes | 0.56 (0.38-0.81) |  | 0.76 (0.56-1.02) |  | 1.13 (0.64-2.00) |  | 0.79 (0.56-1.11) |  |
| Interval time |  | 0.661 |  | 0.974 |  | 0.919 |  | 0.859 |
| < 60 | 1.00 |  | 1.00 |  | 1.00 |  | 1.00 |  |
| ≥ 60 | 1.10 (0.72-1.67) |  | 1.01 (0.73-1.39) |  | 1.03 (0.57-1.88) |  | 1.04 (0.71-1.51) |  |
| Surgical procedure |  | 0.001 |  | 0.001 |  | 0.302 |  | 0.084 |
| APR | 1.00 |  | 1.00 |  | 1.00 |  | 1.00 |  |
| LAR | 0.94 (0.62-1.42) | 0.771 | 0.83 (0.60-1.15) | 0.264 | 0.67 (0.37-1.22) | 0.187 | 0.90 (0.63-1.30) | 0.576 |
| Hartmann | 2.80 (1.56-5.03) | 0.001 | 2.24 (1.37-3.66) | 0.001 | 1.34 (0.47-3.77) | 0.586 | 1.85 (1.00-3.40) | 0.049 |
| Adjuvant chemotherapy; |  | 0.355 |  | 0.442 |  | 0.364 |  | 0.634 |
| No | 1.00 |  | 1.00 |  | 1.00 |  | 1.00 |  |
| Yes | 1.53 (0.62-3.75) |  | 1.28 (0.68-2.43) |  | 1.92 (0.47-7.90) |  | 1.19 (0.58-2.43) |  |
| Differentiation grade |  | < 0.001 |  | < 0.001 |  | 0.003 |  | 0.003 |
| Low | 1.00 |  | 1.00 |  | 1.00 | . | 1.00 |  |
| Middle | 0.27 (0.16-0.44) | .000 | 0.39 (0.26-0.58) | < 0.001 | 0.34 (0.17-0.67) | 0.002 | 0.46 (0.29-0.73) | 0.001 |
| High | 0.20 (0.05-0.85) | .029 | 0.48 (0.20-1.14) | 0.095 | 0.74 (0.21-2.55) | 0.629 | 0.34 (0.10-1.11) | 0.073 |
| Unknown | 0.41 (0.26-0.64) | < 0.001 | 0.44 (0.30-0.64) | < 0.001 | 0.31 (0.16-0.63) | 0.001 | 0.48 (0.30-0.76) | 0.002 |
| TRG |  | 0.247 |  | 0.035 |  | 0.009 |  | 0.103 |
| 0 | 1.00 |  | 1.00 |  | 1.00 |  | 1.00 |  |
| 1 | 1.26 (0.71-2.25) | 0.431 | 1.65 (1.04-2.64) | 0.036 | 2.41 (0.95-6.12) | 0.064 | 1.48 (0.86-2.54) | 0.153 |
| 2 | 1.66 (0.98-2.79) | 0.058 | 1.86 (1.21-2.86) | 0.005 | 1.91 (0.77-4.73) | 0.164 | 1.72 (1.05-2.82) | 0.031 |
| 3 | 1.21 (0.48-3.03) | 0.684 | 2.04 (1.07-3.89) | 0.031 | 5.44 (1.93-15.30) | 0.001 | 2.18 (1.07-4.44) | 0.031 |
| ypT |  | 0.026 |  | 0.065 |  | 0.240 |  | 0.072 |
| T0 | 1.00 |  | 1.00 |  | 1.00 |  | 1.00 |  |
| T1 | 0.57 (0.13-2.44) | 0.448 | 1.16 (0.48-2.81) | 0.744 | 3.61 (1.02-12.79) | 0.047 | 0.95 (0.33-2.76) | 0.928 |
| T2 | 0.94 (0.51-1.73) | 0.851 | 1.51 (0.94-2.42) | 0.086 | 1.99 (0.77-5.12) | 0.155 | 1.20 (0.70-2.05) | 0.514 |
| T3 | 1.68 (1.00-2.83) | 0.052 | 1.87 (1.22-2.89) | 0.005 | 2.46 (1.01-5.97) | 0.047 | 1.81 (1.12-2.93) | 0.016 |
| T4 | 2.19 (1.02-4.71) | 0.046 | 1.72 (0.85-3.47) | 0.130 | 1.39 (0.28-6.86) | 0.690 | 1.13 (0.46-2.80) | 0.786 |
| ypN |  | < 0.001 |  | < 0.001 |  | 0.009 |  | < 0.001 |
| N0 | 1.00 |  | 1.00 |  | 1.00 |  | 1.00 |  |
| N1 | 1.89 (1.21-2.95) | 0.005 | 1.92 (1.36-2.71) | < 0.001 | 1.83 (0.97-3.46) | 0.064 | 2.13 (1.44-3.15) | < 0.001 |
| N2 | 2.82 (1.68-4.73) | < 0.001 | 2.53 (1.66-3.84) | < 0.001 | 2.90 (1.41-5.95) | 0.004 | 2.58 (1.59-4.18) | < 0.001 |
| TD |  | < 0.001 |  | < 0.001 |  | 0.006 |  | < 0.001 |
| negative | 1.00 |  | 1.00 |  | 1.00 |  | 1.00 |  |
| positive | 2.23 (1.48-3.35) |  | 2.06 (1.48-2.87) |  | 2.26 (1.26-4.07) |  | 2.04 (1.40-2.98) |  |
| LNY |  | 0.030 |  | 0.012 |  | 0.293 |  | 0.011 |
| ≤ 11 | 1.00 |  | 1.00 |  | 1.00 |  | 1.00 |  |
| > 11 | 0.64 (0.43-0.96) |  | 0.67 (0.49-0.92) |  | 0.74 (0.42-1.30) |  | 0.63 (0.44-0.90) |  |
| Vascular invasion |  | 0.002 |  | < 0.001 |  | 0.001 |  | < 0.001 |
| negative | 1.00 |  | 1.00 |  | 1.00 |  | 1.00 |  |
| positive | 2.42 (1.40-4.17) |  | 2.47 (1.58-3.85) |  | 3.39 (1.66-6.95) |  | 2.65 (1.61-4.36) |  |
| Neural invasion |  | 0.058 |  | 0.036 |  | 0.830 |  | 0.034 |
| negative | 1.00 |  | 1.00 |  | 1.00 |  | 1.00 |  |
| positive | 1.63 (0.98-2.70) |  | 1.56 (1.03-2.37) |  | 0.90 (0.36-2.27) |  | 1.65 (1.04-2.64) |  |
| CRM invasion |  | 0.025 |  | 0.200 |  | 0.002 |  | 0.607 |
| negative | 1.00 |  | 1.00 |  | 1.00 |  | 1.00 |  |
| positive | 4.94 (1.22-20.06) |  | 2.49 (0.62-10.04) |  | 9.61 (2.34-39.51) |  | 0.05 (< 0.001, 4620) |  |

Note: ^#^ All HRs (95% CI) were calculated through univariate Cox regression models.

Abbreviation: HR, hazard ratio; LNY, lymph node yield; TD, tumor deposit; TRG, tumor regression grade; CRM, circumferential resection margin；OS, overall survival; DFS, disease free survival; LRFS, local recurrence free survival; DMFS, distant metastasis free survival.

**Supplemental Table 2. Multivariate Cox analyses in relation to survival times**

| Characteristics | | OS | | DFS | | LRFS | | DMFS | |
| --- | --- | --- | --- | --- | --- | --- | --- | --- | --- |
|  | | HR^#^ (95% CI) | P | HR^#^ (95% CI) | P | HR^#^ (95% CI) | P | HR^#^ (95% CI) | P |
| cT | |  | 0.035 |  | 0.029 |  | 0.008 |  | 0.287 |
| T2 | | 1.00 |  | 1.00 |  | 1.00 |  | 1.00 |  |
| T3 | | 0.87 (0.20-3.76) | 0.849 | 1.80 (0.43-7.50) | 0.422 | 4524  (< 0.01, > 200) | 0.920 | 2.39 (0.32-17.58) | 0.394 |
| T4 | | 1.61 (0.35-7.33) | 0.539 | 2.83 (0.66-12.12) | 0.161 | 12404  (< 0.01, > 200) | 0.910 | 3.18 (0.42-24.08) | 0.263 |
| cN | |  | 0.565 |  | 0.442 |  | 0.368 | . | 0.728 |
| N0 | | 1.00 |  | 1.00 |  | 1.00 |  | 1.00 |  |
| N1 | | 1.45 (0.69-3.05) | 0.334 | 1.35 (0.78-2.34) | 0.285 | 1.32 (0.43-4.06) | 0.628 | 1.08 (0.58-2.00) | 0.805 |
| N2 | | 1.50 (0.70-3.21) | 0.294 | 1.45 (0.82-2.56) | 0.202 | 1.95 (0.63-6.03) | 0.247 | 1.24 (0.66-2.33) | 0.505 |
| Interval chemotherapy | |  | 0.662 |  | 0.386 |  | 0.541 | . | 0.505 |
| No | | 1.00 |  | 1.00 |  | 1.00 |  | 1.00 |  |
| Yes | | 0.44 (1.00-0.44) |  | 0.87 (0.63-1.19) |  | 1.21 (0.66-2.24) |  | 0.88 (0.61-1.28) |  |
| Surgical procedure | |  | 0.007 |  | 0.001 |  | 0.543 | . | 0.091 |
| APR | | 1.00 |  | 1.00 |  | 1.00 |  | 1.00 |  |
| LAR | | 0.92 (0.59-1.45) | 0.724 | 0.85 (0.60-1.20) | 0.355 | 0.79 (0.42-1.52) | 0.484 | 0.96 (0.65-1.41) | 0.830 |
| Hartmann | | 2.61 (1.36-5.01) | 0.004 | 2.33 (1.36-3.99) | 0.002 | 1.48 (0.48-4.53) | 0.496 | 1.98 (1.03-3.82) | 0.041 |
| Differentiation grade |  | | 0.001 |  | 0.298 |  | 0.231 | . | 0.130 |
| Low | | 1.00 |  | 1.00 |  | 1.00 | . | 1.00 |  |
| Middle | | 0.33 (0.19-0.57) | < 0.001 | 0.49 (0.32-0.77) | 0.002 | 0.45 (0.20-1.00) | 0.051 | 0.57 (0.34-0.94) | 0.027 |
| High | | 0.30 (0.07-1.35) | 0.117 | 0.62 (0.24-1.57) | 0.313 | 0.69 (0.17-2.78) | 0.598 | 0.41 (0.12-1.43) | 0.159 |
| Unknown | | 0.60 (0.34-1.07) | 0.085 | 0.67 (0.41-1.07) | 0.096 | 0.49 (0.20-1.19) | 0.114 | 0.73 (0.42-1.27) | 0.269 |
| TRG | |  | 0.558 |  | 0.298 |  | 0.019 | . | 0.011 |
| 0 | | 1.00 |  | 1.00 |  | 1.00 |  | 1.00 |  |
| 1 | | 3.88 (0.49-30.79) | 0.200 | 6.58 (0.68-63.52) | 0.104 | 2.51 (0.17-37.54) | 0.506 | 21.48 (3.00-153.73) | 0.002 |
| 2 | | 3.22 (0.40-26.23) | 0.274 | 6.11 (0.62-60.01) | 0.120 | 1.81 (0.11-29.05) | 0.674 | 21.31 (2.85-159.29) | 0.003 |
| 3 | | 2.86 (0.30-27.03) | 0.360 | 8.27 (0.79-86.35) | 0.077 | 6.80 (0.40-117.02) | 0.187 | 33.56 (4.12-273.10) | 0.001 |
| ypT | |  | 0.560 |  | 0.698 |  | 0.551 | . | 0.062 |
| T0 | | 1.00 |  | 1.00 |  | 1.00 |  | 1.00 |  |
| T1 | | 0.20 (0.02-2.46) | 0.211 | 0.21 (0.02-2.36) | 0.208 | 1.61 (0.09-28.48) | 0.746 | 0.06 (0.01-0.49) | 0.009 |
| T2 | | 0.30 (0.04-2.50) | 0.268 | 0.25 (0.03-2.49) | 0.238 | 0.77 (0.05-12.57) | 0.857 | 0.06 (0.01-0.44) | 0.005 |
| T3 | | 0.40 (0.05-3.33) | 0.396 | 0.22 (0.02-2.21) | 0.198 | 0.57 (0.03-9.72) | 0.695 | 0.06 (0.01-0.47) | 0.007 |
| T4 | | 0.50 (0.05-4.64) | 0.539 | 0.19 (0.02-2.11) | 0.178 | 0.33 (0.01-8.38) | 0.503 | 0.04 (0.004-0.34) | 0.003 |
| ypN | |  | 0.242 |  | 0.098 |  | 0.708 | . | 0.079 |
| N0 | | 1.00 |  | 1.00 |  | 1.00 |  | 1.00 |  |
| N1 | | 1.45 (0.86-2.45) | 0.159 | 1.52 (1.01-2.29) | 0.043 | 1.25 (0.57-2.74) | 0.573 | 0.03 (1.65-1.05) | 0.031 |
| N2 | | 1.68 (0.85-3.31) | 0.135 | 1.57 (0.91-2.70) | 0.108 | 1.50 (0.56-3.99) | 0.418 | 0.11 (1.69-0.89) | 0.107 |
| TD | |  | 0.014 |  | 0.008 |  | 0.038 | . | 0.095 |
| negative | | 1.00 |  | 1.00 |  | 1.00 |  | 1.00 |  |
| positive | | 1.77 (1.12-2.80) |  | 1.64 (1.14-2.36) |  | 2.07 (1.04-4.13) |  | 1.43 (0.94-2.19) |  |
| LNY | |  | 0.003 |  | 0.002 |  | 0.093 | . | 0.005 |
| ≤ 11 | | 1.00 |  | 1.00 |  | 1.00 |  | 1.00 |  |
| > 11 | | 0.52 (0.34-0.80) |  | 0.60 (0.43-0.82) |  | 0.60 (0.33-1.09) |  | 0.58 (0.40-0.85) |  |
| Vascular invasion | |  | 0.807 |  | 0.320 |  | 0.104 | . | 0.325 |
| Negative | | 1.00 |  | 1.00 |  | 1.00 |  | 1.00 |  |
| Positive | | 1.08 (0.57-2.06) |  | 1.32 (0.77-2.27) |  | 2.10 (0.86-5.12) |  | 1.35 (0.74-2.47) |  |
| Neural invasion | |  | 0.925 |  | 0.781 |  | 0.221 | . | 0.637 |
| Negative | | 1.00 |  | 1.00 |  | 1.00 |  | 1.00 |  |
| Positive | | 0.97 (0.55-1.74) |  | 1.07 (0.67-1.72) |  | 0.52 (0.18-1.49) |  | 1.13 (0.67-1.91) |  |
| CRM invasion | |  | 0.068 |  | 0.263 |  | 0.001 | . | 0.964 |
| negative | | 1.00 |  | 1.00 |  | 1.00 |  | 1.00 |  |
| positive | | 4.21 (0.90-19.78) |  | 2.32 (0.53-10.16) |  | 19.50 (3.46-110.02) |  | < 0.01  (< 0.01,> 200) |  |

Note: ^#^Multivariate Cox regression model controlling for cT, cN, interval chemotherapy, surgical procedure, tumor grade, TRG score, ypT, ypN, TD, vascular invasion, neural invasion and CRM.

Abbreviation: HR, hazard ratio; LNY, lymph node yield; TD, tumor deposit; TRG, tumor regression grade; CRM, circumferential resection margin；OS, overall survival; DFS, disease free survival; LRFS, local recurrence free survival; DMFS, distant metastasis free survival.
